# Comprehensive target geometric errors and margin assessment in stereotactic partial breast irradiation

**DOI:** 10.1186/s13014-017-0889-6

**Published:** 2017-09-11

**Authors:** Xin Zhen, Bo Zhao, Zhuoyu Wang, Robert Timmerman, Ann Spangler, Nathan Kim, Asal Rahimi, Xuejun Gu

**Affiliations:** 1 Department of Radiation Oncology, The University of Texas Southwestern Medical Center, Dallas, TX 75390 USA; 2 Department of Biomedical Engineering, Southern Medical University, Guangzhou, Guangdong 510515 China; 3 Department of Epidemiology, Biostatistics and Occupational Health, McGill University, 805 Sherbrooke Street West, Montreal, Quebec H3A 0G4 Canada

**Keywords:** Stereotactic partial breast irradiation, Cyberknife, Fiducial, Margin

## Abstract

**Background:**

Recently developed stereotactic partial breast irradiation (S-PBI) allows delivery of a high biologically potent dose to the target while sparing adjacent critical organs and normal tissue. With S-PBI tumoricidal doses, accurate and precise dose delivery is critical to achieve high treatment quality. This study is to investigate both rigid and non-rigid components of target geometric error and their corresponding margins in S-PBI and identify correlated clinical factors.

**Methods:**

Forty-three early-stage breast cancer patients with implanted gold fiducial markers were enrolled in the study. Fiducial positions recorded on the orthogonal kV images on a Cyberknife system during treatment were used to estimate intra-fraction errors and composite errors (including intra-fraction errors and residual errors after patient setup). Both rigid and non-rigid components of intra-fraction and composite errors were analyzed and used to estimate rigid and non-rigid margins, respectively. Univariate and multivariate linear regressions were conducted to evaluate correlations between clinical factors and errors.

**Results:**

For the study group, the intra-fraction rigid and non-rigid errors are 2.0 ± 0.6 mm and 0.3 ± 0.2 mm, respectively. The composite rigid and non-rigid errors are 2.3 ± 0.5 mm and 1.3 ± 0.8 mm, respectively. The rigid margins in the left-right, anterior-posterior, and superior-inferior directions are estimated as 2.1, 2.4, and 2.3 mm, respectively. The estimated non-rigid margin, assumed to be isotropic, is 1.7 mm. The outer breast quadrants are more susceptible to composite errors occurrence than the inner breast quadrants. The target to chest wall distance is the clinical factor correlated with target geometric errors.

**Conclusions:**

This is the first comprehensive analysis of breast target geometric rigid and non-rigid errors in S-PBI. Upon the estimation, the non-rigid margin is comparable to rigid margin, and therefore should be included in planning target volume as it cannot be accounted for by the Cyberknife system. Treatment margins selection also need to consider the impact of relevant clinical factor.

**Electronic supplementary material:**

The online version of this article (10.1186/s13014-017-0889-6) contains supplementary material, which is available to authorized users.

## Background

Accelerated partial breast irradiation (APBI) is an effective alternative to standard whole breast irradiation (WBI) in selected early-stage breast cancer patients undergoing breast conservation therapy [1–6]. Recently developed stereotactic partial breast irradiation (S-PBI) allows 1–5 treatment fractions by delivering a high biologically potent dose to the target, while sparing adjacent critical organs and normal tissue [1, 7–9]. With S-PBI tumoricidal doses, accurate and precise dose delivery is critical to achieve high treatment quality.

The primary challenge in accurate and precise dose delivery is treatment volume definition [10–14], defining regions clinically at risk and accounting for setup errors and intra-fractional motion uncertainties. In this report, we are concerned with the later. In breast irradiation, the soft and deformable nature of breast tissue makes setup and tracking of the breast targets particularly challenging. Inter-fractional setup errors, caused by daily setup variations, are difficult to control. Although the rigid component can be mostly corrected with couch maneuvers prior to beam on, the non-rigid component currently has no effective method to be compensated. Intra-fractional motion, including both respiratory motion and patient movement, is unconscious and unlikely to be eliminated. Intra-fractional errors must be monitored ideally in real-time or close to real-time to justify small planning target volume (PTV) margins. Rigid components of the intra-fractional error can be corrected using robotic delivery system (Cyberknife); however, the non-rigid components cannot be corrected.

An institutional review board (IRB) approved phase-I S-PBI clinical trial using Cyberknife® (Accuray Incorporated, Sunnyvale, CA, USA) [9] was initiated at our institution in 2010. Enrolled patients were treated with fiducial markers, implanted near the target as target surrogates and monitored by orthogonal kV images every minute during beam delivery. The purpose of this study is to analyze breast target geometric errors, both rigid and non-rigid, using these recorded fiducial positions and estimate treatment margins. Furthermore, clinical factors correlated to target geometric errors were investigated with univariate and multivariate analysis.

## Methods

### Patients and fiducial marker placement

Forty-three patients were randomly selected from the cohort enrolled at our institutional clinical trial, which is a single-arm, prospective 5-fraction dose escalation stereotactic radiotherapy study conducted on Cyberknife system. For each enrolled patient, four to five gold fiducial markers (CIVCO Medical Solutions, Orange City, IA) were systematically implanted at least 2-cm apart at the edge of the lumpectomy cavity [15]. Each gold fiducial marker was 3-mm in length and 1.2-mm in diameter. These strategically placed fiducial markers were meant to serve as surrogates for the tumor bed itself to localize targets and track target motion.

### Treatment simulation and planning

Computer tomography (CT) simulation was conducted on each enrolled patient to obtain three-dimensional (3D) anatomic images for treatment planning. During simulation, the patients were set in a supine position with both arms above the head and immobilized by a Vac Loc® bag secured in an immobilization frame. CT scans were started either at or above the mandible, and were extended several centimeters below the inframammary fold (including the entire lung) with a 1.5-mm axial slices spacing. The CT images were imported into the CyberKnife MultiPlan® treatment planning system for target delineation and treatment planning. The clinical target volume (CTV) was defined by uniformly expanding the lumpectomy cavity volume by 10 mm. The PTV was defined as the CTV plus a 5.0-mm margin by excluding the chest wall, the pectoralis muscles, and the region within 5.0-mm distance to the skin. Critical structures were also delineated, such as the heart, the lung, the ipsilateral and contralateral whole breast, and etc. Fiducials identified on the CT images were projected on digitally reconstructed radiographs (DRRs) for subsequent use during treatment set up.

### Treatment delivery, target localization, and tracking

**Fig. 1:**
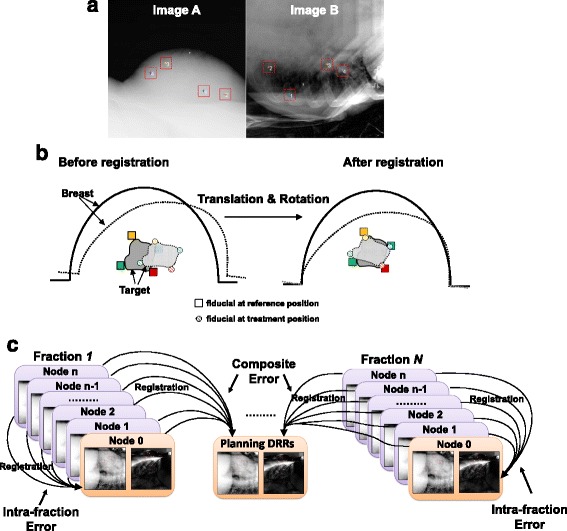
**a** Paired orthogonal kV X-ray images acquired during S-PBI treatment. The red squares indicate the identified fiducials. **b** Illustration of rigid and non-rigid errors. Rigid error is calculated with rigid registration. Non-rigid error accounts for absolute fiducial residual distance after rigid registration. Non-rigid error of a patient is estimated by averaging residual distances over the implanted fiducials. **c** Illustration of intra-fraction error and composite error

Daily target positioning before the delivery of each radiation fraction was achieved by aligning the position of the fiducials on two orthogonally acquired x-ray images to their reference positions on the DRRs derived from the planning CT. Treatment delivery was supported by the Cyberknife Synchrony® Respiratory Tracking System. A typical S-PBI takes about 40 min or more, including patient positioning (~5 mins), robot positioning (~20–30 min) to deliver multiple non-coplanar beams, image acquisition and motion modeling (~5–10 min for an entire fraction treatment), and beam delivery (~7 mins beam-on time). The internal fiducial positions identified on the paired orthogonal x-ray images (Fig. 1a) were correlated with external optical marker positions to establish a respiratory correlation model. Paired orthogonal x-ray images were acquired before treatment delivery to build a correlation model and throughout the treatment delivery to continually verify the model every minute and update if needed. The Cyberknife robotic arm then dynamically moved the beam during delivery to account for the respiration motion based on this correlation model [16]. The captured paired x-ray image sequences were recorded and used to assess target geometric errors.

### Analysis

#### Data structure

Depending on the duration of fractional treatment delivery, 7–73 pairs (with average 29) of x-ray images (512 × 512 with a resolution of 0.4*mm* × 0.4*mm*), called image pairs at *node 0, 1*…) were acquired. *Node 0* refers to the images acquired right after fiducials (the target) was aligned and before the first beam delivery. There were 6105 nodes and 215 fractions for 43 patients (5 fractions for each patient). Statistical analysis was performed at different data levels, including *node*, *fraction,* and *patient levels*. The node level was analyzed from the data extracted from the images at each node; the fraction level was conducted on the data averaged over the nodes in each fraction, and the patient level was performed on the data averaged over the nodes of each patient. The patient level data was used to estimate margin and study its correlation to clinical factors.

#### Error analysis

In the longitudinal time domain, breast target geometric errors can be defined with different reference target positions. *Intra-fraction error* is defined as the target geometric deviation between the target position at *node 0* of each treatment fraction, and subsequent treatment target position (Fig. 1c). *Composite error* is defined as the target geometric deviation between the planning CT and each treatment *node 0*, *node 1*, …, *node n*. In the time domain, the composite error includes both intra-fraction error after treatment start and inter-fraction residual setup errors after initial kV-kV alignment. In the spatial domain, breast target geometric errors (called *total errors*) consist of *rigid* and *non-rigid* errors. Non-rigid error is noted when fiducials failed to align in position relative to one another that would reconstitute their relationship to the tumor cavity at simulation. Rigid and non-rigid errors (Fig. 1b) are analyzed separately. Rigid errors were calculated using 3–5 fiducials’ 3D coordinates through a Horn’s quaternion-based 3D point matching algorithm [17], where the 3D fiducial positions were calculated with paired two-dimensional (2D) fiducial coordinates identified on real-time paired x-ray images (Fig. 1a). Specifically, the rigid component of target error was computed as *T* + *R*, where *T* is the translational error amplitude and *R* is the rotation contributed error amplitude. The *translational error amplitude* is given by $T=\sqrt{x^2+{y}^2+{z}^2}$, where *x*, *y*, and *z* are the translation errors in the Left-Right (LR), Anterior-Posterior (AP), and Superior-Inferior (SI) direction. We used the following equation $R=\sqrt{2}s\sqrt{\varnothing^2+{\theta}^2+{\varphi}^2}$ proposed in [18] to convert the contribution of angular rotation ∅ (roll), *θ*(yaw), and *φ*(pitch) to the error amplitude, where *s* is the radius of the target. In this study, we chose the largest eligible tumor size *s* = 30 *mm*. Non-rigid errors are the fiducial point residual errors after rigid registration. The amplitude of non-rigid error is estimated by averaging all the *k* individual fiducial residual motions (∆*x*_*k*_, ∆*y*_*k*_, ∆*z*_*k*_) after rigid registration, calculated as $N=1/k\sum_k\sqrt{\Delta {x}_k+\Delta {y}_k+\Delta {z}_k}$. The total error is therefore the combination of the above rigid and non-rigid errors, given by *T* + *R* + *N*. The 2D-3D fiducial coordinate conversion is described in detail in Additional file 1: Appendices A. Note that considering the negligible fiducial migration (mean 0 mm) in breast reported in previous study [11], fiducial migration was ignored in this study.

#### Margin estimation

Target margins are derived from composite errors and should account for rigid and non-rigid components. Calculations of the *rigid margin*, the *non-rigid margin* and the *total margin* are detailed in Additional file 1: Appendix B.

#### Impact on breast target geometric errors

Breast target geometric errors may be affected by a number of clinical factors: ① target location; ② mean CT number of breast (CTB, in Hounsfield Units (HUs), indicator of breast density); ③ breast volume (BV, in cm^3^); ④ distance of the target centroid to the chest wall (Dchest, in mm); ⑤ distance of the target centroid to the skin (Dskin, in mm) (④ and ⑤ are indicators of influence from breathing motion); ⑥ PTV-to-breast volume ratio (PBR, relative tumor size); ⑦ ipsilateral breast side (left or right), and ⑧ patient age (PA).

**Fig. 2:**
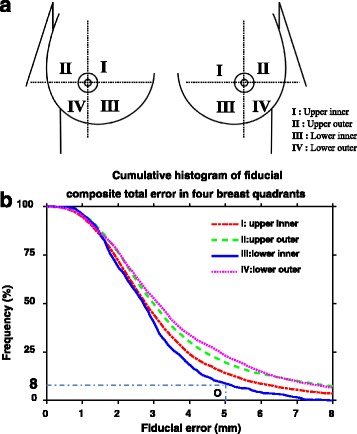
**a** Breast quadrants. The quadrant division is centered at the nipple; **b** Cumulative frequency histogram of fiducial composite error in four breast quadrants (similar to cumulative dose volume histogram fashion). Here, we use point O as an example to explain the curve. The point O represents 8% of time lower inner fiducial has an error at least 5.0 mm or greater

To assess the relationship between target geometric errors and location, the breast was segmented into four quadrants: upper inner, upper outer, lower inner, and lower outer (Fig. 2a). Fiducial markers were categorized based on their location with the four quadrants. The composite total error was used to characterize the quadrant target geometric error.

Spearman’s rho was used for the correlation analysis between the listed clinical factors (2–8) and the target geometric errors. The factors found to be significantly correlated to the target geometric errors were further assessed by univariate linear regression. Multivariate linear regression was also performed to predict the target geometric errors with the listed clinical factors (see Additional file 1: Appendix C for details). Statistical computations and univariate linear regression were performed on the SPSS 19.0 software (SPSS Inc., Chicago, IL). Multivariate regression was accomplished using the statistical computing platform R (version 3.3.0) [19] with the ‘rjags’ library [20]. *P*=0.05 indicated statistical significance.

## Results

### Intra-fractional and composite error

The rigid and non-rigid components of intra-fraction and composite errors were evaluated on different data levels. The mean and SD of intra-fraction and composite breast errors are listed in Table 1. For the intra-fraction, rigid error was 2.0–2.2 mm, the non-rigid error was ~0.3 mm, and the total error was 2.3–2.5 mm. Also, 65% of the patients, 59% of the fractions, and 54% of the nodes presented total error greater than 2.0 mm. For the composite, rigid error was 2.3–2.6 mm, non-rigid error was ~1.3 mm, and total error was 3.6–3.9 mm. In 98% of the patients, 95% of the fractions, and 90% of the nodes, the total error was greater than 2.0 mm. For both the intra-fraction and the composite, AP direction was the largest in rigid errors, followed by SI and LR directions. Rotation was small with mean values less than 0.5^0^ in all three directions. Roll rotation was the largest followed by pitch, while yaw was negligible.

**Table 1:** Intra-fraction and composite breast target geometric errors* calculated at patient, fraction, and node levels

	Data level	Rotation(^0^)			Translation (mm)			Rigid (mm)	Non-rigid (mm)	Total (mm)
		Roll	Yaw	Pitch	LR	AP	SI			
Intra-fraction	Patient	0.5 ± 0.3 (1.6)	0.0 ± 0.0 (0.2)	0.2 ± 0.2 (1.2)	0.8 ± 0.3 (1.9)	1.1 ± 0.4 (2.0)	1.0 ± 0.4 (2.3)	2.0 ± 0.6 (3.9)	0.3 ± 0.2 (0.8)	2.3 ± 0.7 (4.5)
	Fraction	0.5 ± 0.7 (4.2)	0.0 ± 0.1 (0.6)	0.2 ± 0.3 (3.1)	0.8 ± 0.6 (2.9)	1.1 ± 0.6 (3.6)	1.0 ± 0.6 (3.5)	2.1 ± 0.9 (6.0)	0.3 ± 0.3 (2.3)	2.3 ± 1.0 (6.6)
	Node	0.5 ± 0.7 (6.7)	0.1 ± 0.1 (1.1)	0.2 ± 0.4 (5.0)	0.8 ± 0.8 (6.9)	1.1 ± 1.0 (8.6)	1.0 ± 1.0 (7.0)	2.2 ± 1.4 (10.8)	0.3 ± 0.3 (3.8)	2.5 ± 1.5 (11.2)
Composite	Patient	0.5 ± 0.3 (1.4)	0.1 ± 0.1 (0.3)	0.3 ± 0.2 (1.0)	1.0 ± 0.3 (1.9)	1.3 ± 0.4 (2.2)	1.1 ± 0.4 (2.5)	2.3 ± 0.5 (3.6)	1.3 ± 0.8 (3.1)	3.6 ± 1.0 (5.8)
	Fraction	0.5 ± 0.6 (3.8)	0.1 ± 0.1 (0.4)	0.3 ± 0.3 (2.8)	1.0 ± 0.6 (3.1)	1.3 ± 0.6 (3.2)	1.1 ± 0.7 (6.2)	2.4 ± 0.9 (6.8)	1.3 ± 1.0 (5.6)	3.7 ± 1.3 (7.7)
	Node	0.5 ± 0.7 (6.9)	0.1 ± 0.1 (0.8)	0.3 ± 0.4 (4.5)	1.0 ± 0.8 (6.5)	1.3 ± 1.0 (6.4)	1.1 ± 1.0 (8.6)	2.6 ± 1.3 (11.5)	1.3 ± 1.0 (6.6)	3.9 ± 1.6 (13.1)

*Numbers in the parentheses indicate the maximum values; rigid, non-rigid, and total errors are reported as amplitudes

### Margin estimation

The rigid margins in the LR, AP, and SI directions were estimated as 2.1, 2.4, and 2.3 mm, respectively, while the non-rigid margin was estimated as 1.7 mm. For Cyberknife-based S-PBI, with the rigid error corrected during beam delivery, the margins could be non-rigid only, 1.7 mm, in all directions. For treatment platform that do not offer frequent intra-fractional error detection and correction (e.g. conventional LINAC based S-PBI), in which both rigid and non-rigid margins need to be considered, the total margins in the LR, AP, and SI directions were 3.8, 4.1, and 4.0 mm, respectively.

### Impact of different clinical factors on errors

The relationship between the composite error (or the composite total error) and the target location in different breast quadrants was plotted using a cumulative frequency illustration, representing smaller error by lower curves (Fig. 2b). The averaged error was 3.3 ± 2.2 mm (0.1 mm to 16.3 mm) in quadrant I, 3.7 ± 2.6 mm (0.1 mm to 20.2 mm) in quadrant II, 2.9 ± 1.4 mm (0.3 mm to 7.9 mm) in quadrant III, and 3.7 ± 2.4 mm (0.1 mm to 13.8 mm) in quadrant IV. These findings implied that the error in the inner breast quadrants was smaller than that in the outer breast quadrants (I vs II and III vs IV, *p* < 0.0001). Smaller error was also seen in lower quadrant III than in upper quadrant I, while lower quadrant IV and upper quadrant II errors were similar (*p* = 0.58).

For the intra-fraction, statistically significant correlations were found between the non-rigid component and predictors Dchest (Spearman coefficient *ρ* = 0.574 , *p* <  < 0.00) and BV (*ρ* = 0.535 , *p* <  < 0.00). No significant correlations were found for either rigid component or total error.

For the composite, a significant correlation was observed between non-rigid component and predictors Dchest (*ρ* = 0.572 , *p* <  < 0.00) and BV (*ρ* = 0.566 , *p* <  < 0.00), and between total error and Dchest (*ρ* = 0.504 , *p* = 0.001) and BV (*ρ* = 0.42 , *p* = 0.005). No significant correlation was found with clinical predictors and rigid component.

**Fig. 3:**
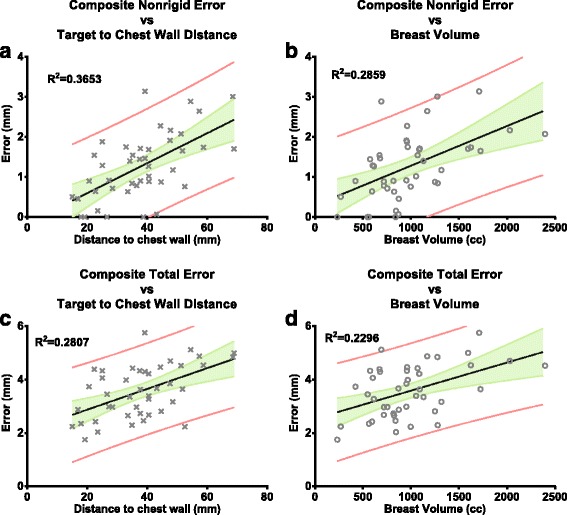
Univariate linear regressions of Dchest (**a** and **c**) and BV (**b** and **d**) with the composite errors, non-rigid component and total. The grey areas indicate the 95% confidence interval and the grey lines encompass the 95% prediction bands

Univariate linear regressions of Dchest and BV with the composite errors, non-rigid component and total, are illustrated in Fig. 3. Although errors increased with increased Dchest and BV, the 95% prediction bands allowed a large range of motion prediction. The quality of the fits was moderate, as indicated by *R* ^2^ equal to 0.37 and 0.29 for the composite non-rigid error, and 0.28 and 0.23 for the composite total error, for Dchest and BV, respectively. These results indicate that breast target geometric errors are complex and may be influenced by multiple factors.

To further analyze correlation between the target geometric error and the listed clinical factors (2–8), multivariate linear regression was adopted. The multivariate linear regression showed that only Dchest is statistically significant correlated to composite errors. For the non-rigid component, the estimated Dchest coefficient was 0.03 with a 95% CI of (0.01–0.05); for the total, the estimated coefficient was 0.03 with a 95% CI of (0.01–0.06). We observed that the SDs of the patient-specific random effect (*σ*_1_), the treatment-specific random effect (*σ*_2_), and the error term (*σ*_3_) were respectively estimated (95% CI) as 0.54 (0.42–0.75), 0.44 (0.03–0.74), and 0.57 (0.04–0.78) for the non-rigid error, and as 0.70 (0.49–0.98), 0.50 (0.04–1.05), and 0.84 (0.031.06) for the composite total error.

## Discussion

In this study, we analyzed breast target geometric errors at different data levels (node, fraction, and patient), at different time frames (intra and composite), and with different components (rigid, non-rigid, and total). Upon data analysis, margins were estimated and clinical factors impacting the errors were identified.

Breast target setup error and motion has been studied by other researchers [10, 11]. Yue et al. [10] investigated breast intra-fraction motion using fiducial positions extracted from orthogonal kV images before and after treatment. Park et al. [11] evaluated intra−/inter-fraction respiratory motion and fiducial stability using pre- and post-treatment 4D CT images and daily online MV orthogonal images. Because the kV/MV image pairs cannot be acquired simultaneously on a conventional LINAC, the 3D fiducial positions extracted from the images already imply a level of uncertainty. Moreover, pre- and post-treatment acquisitions cannot capture intra-fractional breast motion. Real-time magnetic resonance image (MRI) has also been reported to track breast motion [21, 22], though MRI can provide superior soft-tissue contrast of the tumor bed, the reported studies are merely focused on intra-fractional motion using 2D MRI images with limited resolution (e.g. 3.5*mm* × 3.5*mm* in [21]). In contrast, the near real-time fiducial positions logged by the Cyberknife kV imaging system offer complete data set on breast target geometric changes during the course of treatment, allowing a comprehensive study of breast target geometric errors.

In contrast to other studies [10, 11, 21, 23, 24] that mainly focused on rigid error, we analyzed the non-rigid component. We found that the non-rigid component of intra-fraction errors is small, with a mean of 0.3 mm on all data levels. These results are not surprising, since diaphragmatic breathing motion is mostly detected in the abdomen, displacing abdominal organs like the liver more than chest wall structures like the breast. Our findings are consistent with those reported in a previous study [10]. The non-rigid component of composite errors has a mean of 1.3 mm, which is mainly caused by breast deformation during daily setup. We used non-rigid component of composite errors to further estimate non-rigid margins and found that they were similar to rigid margins (1.7 mm vs. ~2.3 mm).

The geometric errors investigated in this study are applicable to fiducial-based S-PBI both with and without real-time motion tracking/error correction. The composite total errors essentially represent the variation observed in conventional LINAC-based S-PBI (with image-guidance but without real-time motion tracking/error correction). In contrast, the non-rigid component of composite error represents the residual error after rigid error compensation in S-PBI.

Based on the analysis in different breast quadrants, targets situated in inner quadrants have smaller errors than those in outer quadrants. Targets in lower quadrant were more stable than those in upper quadrants. BV and Dchest were found to be statistically correlated with breast target geometric errors. A larger breast is likely to present larger error because soft tissue breast is highly deformable and susceptible to movement [10]. Notice that BV and Dchest are intrinsically correlated because a larger target to the chest wall distance is usually observed in patients with larger breast volume. Also note that in Spearman correlation analysis, smaller correlation coefficients *ρ* were always obtained for BV, and specifically for composite total error. Upon multivariate analysis, Dchest was the only clinical factor that is statistically significant to geometric errors. Therefore, compared with BV, Dchest is a clinical factor more relevant to breast geometric errors. Additional margin expansion may be needed for patients with tumors located far from the chest wall.

## Conclusions

We comprehensively analyzed breast target geometric errors by using the real-time recorded fiducial marker positions on a Cyberknife system. The analysis results show that non-rigid and rigid errors are comparable. Upon the margin estimation, the non-rigid margin is similar to rigid margin, and therefore should be included in PTV as it cannot be accounted for by the Cyberknife system. In S-PBI, the outer breast quadrants were more susceptible to target geometric errors than the inner breast quadrants during S-PBI. The target to the chest wall distance is the clinical factor that correlated with breast target geometric errors.

## Additional file

Additional file 1: Appendix A. 2D fiducial coordinates to 3D fiducial position conversion. **Appendix B.** Margin calculations. **Appendix C.** Multivariate linear regression model. (DOCX 29 kb)
