# Supplementary material for: Comprehensive target geometric errors and margin assessment in stereotactic partial breast irradiation
Source: Radiat Oncol. 2017 Sep 11;12:151. doi: 10.1186/s13014-017-0889-6 (PMC5594509; doi:10.1186/s13014-017-0889-6)
Supplement: Additional file 1: Appendix A. — 2D fiducial coordinates to 3D fiducial position conversion. Appendix B. Margin calculations. Appendix C. Multivariate linear regression model. (DOCX 29 kb) [file 13014_2017_889_MOESM1_ESM.docx]

**Appendix A. 2D fiducial coordinates to 3D fiducial position conversion**

The 2D fiducial coordinates $(x_{A},y_{A})$ and $\left( x_{B},y_{B} \right)$on the paired x-ray images $A$ and $B$ (Fig.1a) can be identified using an intensity threshold method. The identified coordinates were converted to 3D coordinates (*x*, *y*, *z*) while planning the CT DICOM coordinate system, expressed by the following equation:

| $x={x_{A}\left( D_{A}-d_{b} \right)}/{2L_{A}} +{x_{B}\left( D_{B}-d_{a} \right)}/{2L_{B}}$  $y=\left( d_{a}-d_{b} \right)/\sqrt{2}$  $z=\left( d_{a}+d_{b} \right)/\sqrt{2}$  $d_{a}={D_{A}y_{A}(L_{A}-y_{B})}/{L_{A}L_{B}-y_{A}y_{B}}$; $d_{b}={D_{B}y_{B}(L_{B}-y_{A})}/{L_{A}L_{B}-y_{A}y_{B}}$ | (1) |
| --- | --- |

where $D_{A}$, $D_{B}$ and $L_{A}$, $L_{B}$ define the source-object distance and source-imager distance. Here, source refers to the kV imager source, object is the center defined by the two orthogonal imagers. Imagers are detector planes $A$ and $B$ in the Cyberknife system. $D_{A}$, $D_{B}$ and $L_{A}$, $L_{B}$ values are machine-specific and fixed at the Cyberknife installation.

**Appendix B. Margin calculations**

The margins related to rigid error (called *rigid margin*) were calculated using van Herk’s well-accepted recipe for various disease sites [[1-3](#_ENREF_1)]: $M_{r}=2.5\Sigma+0.7\sigma$, where M_r_ denotes the rigid margin in one translational direction [Anterior-Posterior (AP); Left-Right (LR); Superior-Inferior (SI)]. Σ and σ indicate systematic and random errors, respectively [[1](#_ENREF_1)]. The mean and standard deviation (SD) of the rigid errors (fraction level) are first obtained for each patient. The systematic error Σ is calculated as the SD of the means for each patient, and the random error σ is the root mean square of the SDs of all patients. Non-rigid errors are mainly affected by breast deformation. We propose to estimate the margin related to non-rigid errors (called *non-rigid margin*) in one translational direction as$M_{\mathrm{nr}}=\frac{2\delta}{\sqrt{3}}, \mathrm{with} \delta=\sqrt{\frac{\sum_{i}^{A} N_{i}^{2}}{A}}$, where A indicates the number of patients; $N_{i}$is the *i*th patient’s mean non-rigid error. In this formula, we assume that non-rigid errors are randomly directed in 3D space and the group mean is zero. Also, with a zero mean, $\delta$ is essentially a SD and $2\delta$ provides a 95% confidence interval (CI) if we assume that non-rigid errors follow a normal distribution. Finally, the factor $\frac{1}{\sqrt{3}}$ converts 3D amplitude to one direction (AP, LR, and SI) by assuming each direction equally contributed. Overall, the *total margin* in one translational direction can be written as $M=M_{r}+M_{\mathrm{nr}}$.

**Appendix C. Multivariate linear regression model**

Given the hierarchical structure of our patient data, multivariate linear regression was described as a multi-level modelling problem, where patient-specific random effect and treatment fraction-specific random effect on the prediction results were allowed. Multivariate linear regression was formulated as:

| $\vec{\Delta}_{ijk}=\beta_{0}+\sum_{m=1}^{7} \beta_{m}P_{ijk}^{m}+\mu_{i}+\mu_{j}+\epsilon_{ijk}$ | (2) |
| --- | --- |

where $\vec{\Delta}_{ijk}$ was the target geometric error from the $kth$ measurement in the $jth$ treatment fraction of patient $ith$, $\beta_{0}$ was the regression intercept, $\beta_{1}$ to $\beta_{7}$were the regression coefficients of the seven clinical predictors $P_{ijk}^{1}$ to $P_{ijk}^{7}$ (i.e. the CTB, BV, Dlung, Dskin, PBR, LR and PA respectively). $\mu_{i}$ and $\mu_{j}$ were the random components of the patient-specific effect and treatment fraction-specific effect, which follow normal distributions $\mu_{i}\sim N\left( 0,\sigma_{1}^{2} \right)$and $\mu_{j}\sim N\left( 0,\sigma_{2}^{2} \right)$. $\epsilon_{ijk}$ was the regression error with distribution $\epsilon_{ijk}\sim N\left( 0,\sigma_{3}^{2} \right)$. The model was fitted using the Bayesian method [[4](#_ENREF_4), [5](#_ENREF_5)].

**References**

1. van Herk M: **Errors and margins in radiotherapy.** *Seminars in Radiation Oncology* 2004, **14:**52-64.

2. Ecclestone G, Bissonnette JP, Heath E: **Experimental validation of the van Herk margin formula for lung radiation therapy.** *Med Phys* 2013, **40:**111721.

3. Gupta T, Chopra S, Kadam A, Agarwal JP, Devi PR, Ghosh-Laskar S, Dinshaw KA: **Assessment of three-dimensional set-up errors in conventional head and neck radiotherapy using electronic portal imaging device.** *Radiat Oncol* 2007, **2:**44.

4. Brown PJ, Vannucci M, Fearn T: **Multivariate Bayesian Variable Selection and Prediction.** *Journal of the Royal Statistical Society Series B (Statistical Methodology)* 1998, **60:**627-641.

5. O'Hara RB, Sillanpaa MJ: **A review of Bayesian variable selection methods: what, how and which.** 2009**:**85-117.
